# Enhancing Polymer Composite Thermal Conductivity by Liquid Metal and Carbon Fiber Dual Fillers

**DOI:** 10.3390/polym18151822

**Published:** 2026-07-25

**Authors:** Zhao Zhao, Yiwei Geng, Jingliang Li, Juan Zhang, Shuaifei Zhao

**Affiliations:** 1 Institute for Frontier Materials (IFM), Deakin University, Geelong, VIC 3216, Australiajingliang.li@deakin.edu.au (J.L.); 2 Institute for Polymer Composites, Jiangsu University of Technology, Changzhou 213001, China

**Keywords:** poly(vinylidene fluoride), carbon fibers, eutectic gallium–indium liquid metals, polymer composites, thermal conductivity

## Abstract

Polymers, including poly(vinylidene fluoride) (PVDF), have been widely used in daily life and industries, such as in membrane filtration, food packing and heat exchanging. However, pristine polymers suffer from inherently low thermal conductivity. Herein, thermally conductive poly(vinylidene fluoride) (PVDF) composites were prepared by incorporating eutectic gallium–indium (EGaIn) liquid metals (LMs) and carbon fibers (CFs) as dual fillers. The effects of the LMs and the CFs on the thermal and mechanical properties of the PVDF composites were characterized via thermal conductivity, morphology, crystallinity, and mechanical testing. The CFs improved the dispersion of the LM droplets within the PVDF matrix, forming continuous thermally conductive pathways, thereby enhancing the composite thermal conductivity. A maximum thermal conductivity of 0.40 W/(m·K), an increase of 154% over the pure PVDF, was achieved when 1 wt% CF was added to the PVDF composites containing 5 wt% LMs. Mechanical testing showed that the CFs compensated for the slight reduction in tensile strength due to the LM addition, yielding a tensile strength of 30 ± 3 MPa, slightly higher than that of the pure PVDF. Characterization analyses confirmed that the crystalline and chemical structure of the PVDF matrix were preserved after the filler addition, indicating minimal alteration of the PVDF matrix. These results suggest that the PVDF/LM/CF composites exhibit a balanced enhancement of the thermal conductivity and mechanical strength, making them potential candidates for thermal management applications. This study provides insights into designing high-performance polymer composites with dual functionality for advanced engineering applications.

## 1. Introduction

Polymers have become essential materials in our daily lives due to their unique properties, such as corrosion resistance [1], light weight [2], and versatile processability [3]. These features make polymers important in a wide range of applications, from consumer goods [4] and packaging [5] to advanced aerospace and biomedical devices [6,7]. However, one major drawback of polymers is their inherently low thermal conductivity (typically < 0.5 W/(m·K) [8]), which limits their potential in heat-dissipating applications. In areas such as electronic components [9], heat exchangers [10], and energy storage devices [11], where efficient heat dissipation is crucial to performance and reliability, the low thermal conductivity of polymers becomes a significant challenge.

To address these limitations, polymer-based composites have emerged as promising candidates for thermal management applications. By incorporating thermally conductive fillers into polymer matrices, it is possible to overcome the inherent low thermal conductivity of polymers while retaining their favorable mechanical and chemical properties [8,12,13]. These composites offer enhanced thermal conductivity along with the advantages of lightweight structures [14], ease of manufacturing [15], and chemical stability [16], making them suitable for advanced applications in industries such as electronics [17], automotive [18], and energy storage [19]. Among polymers, poly(vinylidene fluoride) (PVDF) has been widely used across various industries because of its excellent chemical resistance, thermal stability, and mechanical robustness [20,21,22]. Despite these advantages, the low thermal conductivity of PVDF (~0.17 W/(m·K) [23]) remains a significant limitation for its use in thermal management systems. Therefore, enhancing the thermal conductivity of PVDF is critical to expanding its applicability in the areas that require efficient heat transfer, while maintaining its favorable mechanical and chemical properties.

Several methods have been explored to enhance the thermal conductivity of PVDF, such as hot-pressing, electrospinning, and blending. Hot-pressing is a widely used technique that involves applying heat and pressure to compact the polymer-filler mixture, thereby improving filler dispersion and alignment [24]. For example, Zhou et al. [25] used hot-pressing to prepare PVDF composites with a thermal conductivity of up to 1.74 W/(m·K). Electrospinning can produce highly aligned polymer nanofibers that incorporate thermally conductive fillers along the fiber axes. The aligned structure facilitates anisotropic thermal conductivity, allowing for more efficient heat transfer along specific directions [22]. For example, Zhang et al. [26] fabricated boron nitride/PVDF composite films by electrospinning, achieving a maximum thermal conductivity of 7.29 W/(m·K). These two methods offer significant improvements in thermal conductivity, but each comes with challenges. Hot-pressing may compromise mechanical flexibility, while electrospinning can be time-consuming and may require post-processing steps to stabilize the fibers. Blending thermally conductive fillers into PVDF is one of the simplest and most widely used methods for enhancing thermal conductivity. The key advantages of blending include its simplicity, low cost, and scalability for mass production [27,28,29]. Guo et al. [30] improved the thermal conductivity of PVDF-based nanocomposites to 1.55 W/(m·K) by incorporating functionalized multi-walled carbon nanotubes into PVDF via the blending method.

Incorporating thermally conductive fillers into polymers is an effective strategy to improve their inherently low thermal conductivity. Gallium-based liquid metals (LMs) have attracted considerable attention due to their high thermal conductivity (e.g., Ga, 29 W/(m·K) [31]; and the eutectic gallium–indium alloy (EGaIn) 26 W/(m·K) [32]), high fluidity [33], and low melting points (typically near room temperature [34]). LMs can form interconnected pathways that significantly enhance heat transfer within a polymer matrix. For example, Jia et al. [35] developed a thermally conductive polymer composite film with high in-plane and through-plane thermal conductivity (7.14 and 1.68 W/(m·K), respectively) by integrating LMs with aramid nanofibers to form efficient heat conduction networks. However, the thermal conductivity enhancement is often limited by filler agglomeration and poor interfacial compatibility between the LMs and the polymer matrix [36,37]. Furthermore, the high surface tension (~700 mN/m [38]) and fluidity of LMs make them prone to leak from the polymer matrix [39], which disrupts the thermally conductive pathways and reduces the overall heat transfer efficiency of the polymer matrix.

To address these challenges and further improve the thermal conductivity of polymer composites, combining LMs with other thermally conductive fillers is a promising strategy. For example, Yi et al. [40] developed a polymer composite by incorporating carbon tube-stabilized gallium-based LMs, achieving a through-plane thermal conductivity of 2.19 W/(m·K) along with excellent dispersion stability. Similarly, Chen et al. [41] constructed thermally enhanced thermoplastic elastomer composites by integrating gallium-based LMs with copper powders, reaching a high thermal conductivity of 32.71 W/(m·K). These studies provide solutions to the challenges associated with direct embedment of LMs into polymers for advanced thermal management. Carbon fibers (CFs), with a high aspect ratio and thermal conductivity along the fiber axis (~1000 W/(m·K) [42]), demonstrated significant potential for enhancing heat transfer in polymer composites [43]. Incorporating CF and LM mixtures into PVDF could introduce additional heat transfer pathways and reduce thermal resistance by promoting filler–filler interactions and strengthening the percolation network. Furthermore, CFs can also improve the mechanical strength of polymer composites, offering dual advantages in both thermal and mechanical performance [43].

This study builds upon our previous works on thermally conductive PVDF composites using CFs and hybrid fillers [43,44], where high thermal conductivity was achieved through relatively high filler loadings. In this work, we aim to explore a filler-efficient strategy that maintains effective thermal conductivity enhancement while minimizing the total filler content. This approach addresses the trade-off between thermal performance, mechanical stability, and material cost, providing a practical route for scalable thermal management applications. Here, we systematically investigated the effect of CF incorporation on the thermal conductivity enhancement of PVDF composites containing EGaIn. Two series of PVDF composites were prepared: 5 wt% of EGaIn was added to the PVDF matrix (marked as PVDF/LM-5), followed by the addition of 1 wt% CFs to the PVDF/LM-5 base (marked as 1%CF@PVDF/LM-5). The morphology, thermal conductivity, crystallinity, and mechanical properties of the composites were characterized to evaluate the role of the CFs in promoting thermal conductive pathways and improving composite performance. The findings are expected to provide insights into the design of high-performance thermally conductive PVDF composites, with potential applications in thermal management systems.

## 2. Experimental Section

### 2.1. Materials

PVDF powder (average M_w_ ~534,000) and eutectic gallium–indium (EGaIn, Ga 75.5%, In 24.5%) alloy were purchased from Sigma-Aldrich Pty Ltd. (Sydney, Australia). Pristine milled CF (K223HM, PITCH 90t, fiber diameter of 11 µm, thermal conductivity of 550 W/(m∙K)), without sizing agents, was provided by Mitsubishi Chemical Cooperation (Tokyo, Japan).

Other chemicals included ethanal (undenatured 100% AR, Chem-Supply, Gillman, Australia) and dimethylformamide (DMF) (ChemSupply, Gillman, Australia).

### 2.2. Preparation of PVDF/LM and PVDF/LM/CF Composites

**Figure 1:**
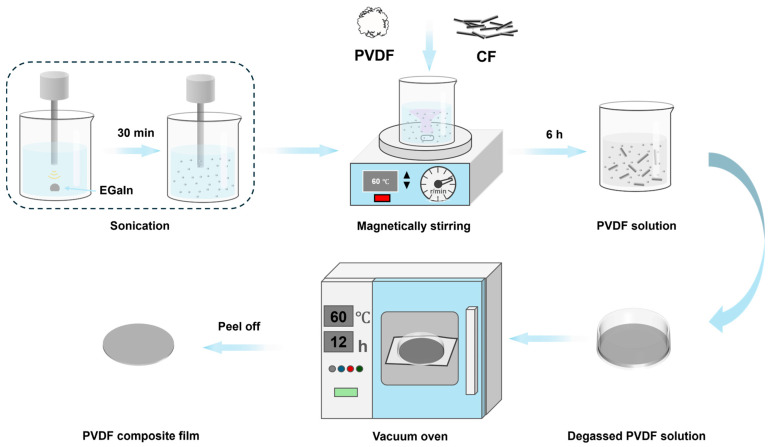
The preparation procedure of the PVDF/LM/CF composites.

The preparation procedure of the PVDF-based composites is demonstrated in Figure 1. A certain amount of EGaIn was dispersed in DMF solvent and sonicated in an ice bath using a horn-based sonicator (Q700 Sonicator, QSonica Sonicator, Newtown, CT, USA) for 30 min at an amplitude of 55 with 3 s pulse and 1 s pulse off. A certain amount of PVDF powder and 1 wt% CFs were added into the EGaIn/DMF mixture and stirred at 60 °C for 6 h until all PVDF was dissolved. The solution was then poured into a glass mold and degassed. The degassed solution was then placed in a vacuum oven at 60 °C overnight, followed by 110 °C for 1 h. The dried film was then peeled off for use. The composite film was named n%CF@PVDF/LM-X, where n is the mass fraction of the CFs added into the PVDF/EGaIn base, and X is the mass fraction of the EGaIn added into the PVDF/EGaIn composite. For example, PVDF/LM-5 indicates 5 wt% of the EGaIn was added into the PVDF matrix, and 1%CF@PVDF/LM-5 means 1 wt% of the CFs was added into the PVDF/LM-5 composites.

### 2.3. Characterization of PVDF/LM/CF Composites

***Micro-structure characterization***. The fractured morphology of the PVDF-based composites was characterized by scanning electron microscopy (SEM) (Phenom ProX Desktop, Thermo Fisher Scientific Inc., Waltham, MA, USA). The composites were firstly immersed in liquid nitrogen until hard and then cracked into two pieces. The surface and the fractured surface were fixed by conductive adhesive tapes and coated with gold.

***Thermal conductivity evaluation***. The thermal conductivity (*λ*, W/(m·K)) was calculated by

*λ*(*T*) = *α*(*T*) × *C_p_*(*T*) × *ρ*(*T*) (1)

where α is the thermal diffusivity (mm^2^/s), *C_p_* is the specific heat capacity (J/(kg·K)), and *ρ* is the density (kg/m^3^). These parameters are the function of temperature (*T*). The *α* of the composites was obtained using a Laser Flash Analyser (LFA 457, NETZSCH, Selb, Germany). The sample was trimmed to 12.7 mm in diameter to fit the sample holders. The data of each sample was recorded three times with a time interval of 3 min at room temperature. *C_p_* was determined by differential scanning calorimetry (DSC) (TA Q200, TA Instruments, New Castle, DE, USA) with a heating rate of 30 °C/min, and its value at room temperature (25 °C) was extracted for thermal conductivity calculation. *ρ* was calculated based on the sample weight and volume.

***Crystallinity and crystal structure characterization***. The melting and crystallization behaviors of the PVDF-based composites were investigated by differential scanning calorimetry (DSC) (Discovery 25, TA Instruments, New Castle, DE, USA). The mass of each sample was about 5–10 mg. All the samples were set under a “heating-cooling-heating” cycle under a continuous nitrogen flow of 50 mL/min: heating from room temperature to 180 °C at 20 °C/min, then maintaining for 5 min to avoid the thermal history, and then cooling to 80 °C at 10 °C/min, followed by reheating to 180 °C at 10 °C/min.

The crystallinity degree (*X*_c_) of the PVDF matrix was calculated by:

(2) $$X_{c}=\frac{{∆H}_{m}}{{\emptyset \times ∆H}_{m}^{0}}\times 100\%$$

where ${∆H}_{m}$ is the fusion enthalpy of the samples from the DSC heating scan, ${∆H}_{m}^{0}$ is the fusion enthalpy of the completely crystalline PVDF (104.6 J/g [45]), and ∅ is the relative mass fraction of PVDF in the composite (%).

The X-ray diffraction (XRD) patterns of the PVDF-based composites were obtained by a diffractometer (X’Pert Powder, Malvern Panalytical, Malvern, UK) with Cu Kα (*λ* = 1.5418 Å) radiation at the condition of 40 kV and 30 mA at room temperature.

***Chemical property evaluation***. A Fourier transform infrared spectroscopy (FTIR) spectrometer (Bruker, Ettlingen, Germany) with attenuated total reflectance (ATR-FTIR) mode was used to evaluate the chemical properties of the PVDF-based composites. The FTIR spectra were recorded in the wavenumber range of 400–4000 cm^−1^ with a resolution of 4 cm^−1^.

***Mechanical property characterization***. Tensile tests of the PVDF-based composites were obtained on Instron 5567 (Instron, Norwood, MA, USA). Each sample was trimmed to rectangles with width of 4 mm and length of 30 mm by a self-made mold. Each sample was pulled at a constant rate of 10 mm/min until fracturing. The strain of the polymer composite was evaluated by the internal crosshead displacement of the machine. The testing result was averaged on repeating three samples.

## 3. Results and Discussion

### 3.1. Morphology of PVDF-Based Composites

**Figure 2:**
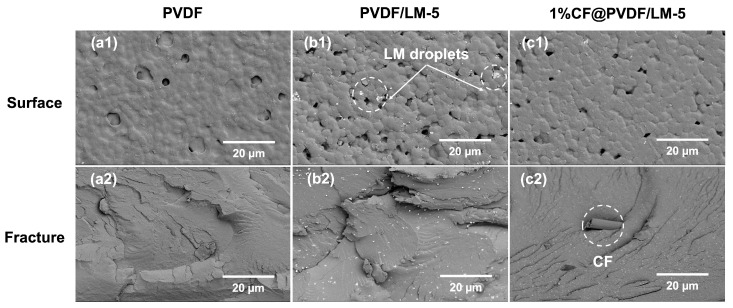
The surface (**a1**–**c1**) and fracture morphologies (**a2**–**c2**) of the pure PVDF, PVDF/LM-5, 1%CF@PVDF/LM-5 composites, respectively.

Figure 2 reveals the SEM images of the distinct surface and fracture morphologies of the PVDF, PVDF/LM-5, and 1%CF@PVDF/LM-5 composites. The surface morphology of the pure PVDF (Figure 2(a1)) showed a smooth, homogeneous structure, confirming the uniformity of the PVDF phase. In the PVDF/LM-5 composite (Figure 2(b1)), the dispersed LM droplets were visible within the PVDF matrix. These droplets were relatively large, indicating agglomerations of the LMs. This may be due to the poor compatibility and surface tension mismatch between the PVDF matrix and the LMs, leading to droplet agglomerations.

In contrast, the 1%CF@PVDF/LM-5 composite (Figure 2(c1)) qualitatively appeared to exhibit a smaller LM droplet size compared to the PVDF/LM-5 composite (Figure 2(b1), no quantitative image analysis was performed). This reduction in droplet size likely resulted from the presence of the CFs, which may act as nucleation points, promoting a more refined and evenly distributed droplet morphology. The CFs can also help to disperse the LMs by providing additional interaction sites within the PVDF matrix, thereby reducing the tendency for the LM droplets to aggregate. This improved dispersion of the LM droplets is expected to enhance thermal conductivity by establishing more uniform conductive pathways throughout the matrix.

The fracture morphologies (Figure 2(a2–c2)) offer additional insights into the internal structure of the composite. The pure PVDF (Figure 2(a2)) had a relatively smooth fracture surface, indicating a ductile failure typical of a polymer phase. In the PVDF/LM-5 composite (Figure 2(b2)), the fracture surface was more irregular, with visible voids around the LM droplets. These voids likely resulted from the interfacial debonding between the PVDF matrix and the LMs under stress, which can compromise mechanical strength by creating regions prone to crack initiation and propagation.

In the 1%CF@PVDF/LM-5 composite (Figure 2(c2)), the fracture surface showed a networked structure around the CFs. The CFs acted as reinforcing elements, helping with load transfer and crack resistance within the composite, and reducing the formation of large voids observed in the PVDF/LM-5 composite. The reinforcement provided by the CFs not only led to a more uniform distribution of the LM droplets but also improved overall mechanical stability by creating energy-dissipative paths along the fiber interfaces.

### 3.2. Thermal Conductivity

**Figure 3:**
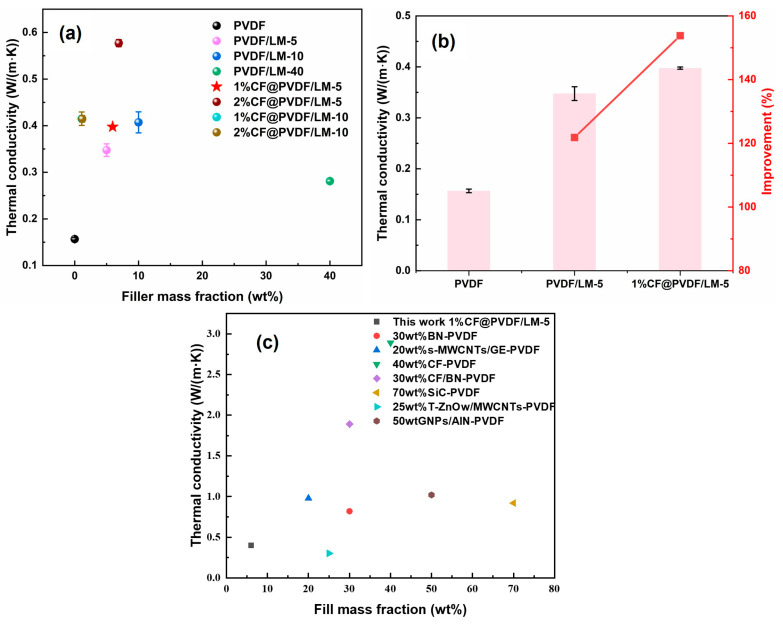
The thermal conductivity of different PVDF-based composites at room temperature: (**a**) the effect of the filler mass fraction, (**b**) thermal conductivity enhancement by the LMs and CFs, and (**c**) a comparison of thermal conductivity of PVDF composites with different fillers.

The thermal conductivity values of the pure PVDF and the PVDF-based composites at room temperature are presented in Figure 3. The pure PVDF showed a baseline thermal conductivity of 0.16 W/(m·K), reflecting the inherently low thermal conductivity of PVDF. After adding 5 wt% LMs in the PVDF matrix, the thermal conductivity increased to 0.35 W/(m·K), doubled that of the pure PVDF (an improvement of 122%). This enhancement is attributed to the high thermal conductivity of the LMs, which introduced localized thermal conductive pathways within the PVDF matrix. However, larger LM droplets (Figure 2(b1)) limited the connectivity across the composite, thus restricting the improvement in thermal conductivity of the composite. Increasing the LMs to 10 wt% (i.e., PVDF/LM-10) further improved the thermal conductivity to 0.41 W/(m·K). However, at 40 wt% LM loading (i.e., PVDF/LM-40), a decrease in thermal conductivity to 0.28 W/(m·K) was observed. This is because the LMs leaked significantly from the PVDF matrix during the fabrication process.

The incorporation of 1 wt% CFs into the PVDF/LM-5 composite increased the thermal conductivity to 0.40 W/(m·K) (an improvement of 154% compared to the pure PVDF), while 2 wt% CFs led to a substantial improvement, reaching 0.58 W/(m·K) (Figure 3a). This is due to the combined effects of the LMs and the CFs. The CFs appeared to act as a bridging element between the dispersed LM droplets, promoting more continuous thermal conductive pathways across the PVDF matrix. Additionally, the CFs can help refine the dispersion of the LM droplets (Figure 2(c1)), reducing gaps and enhancing heat transfer. This combined network of the LMs and the CFs formed an interconnected structure that increased thermal conductivity beyond that of the PVDF/LM-5 alone.

We also compared this performance with previous similar studies based on PVDF-based thermal conductive systems. In our previous study, when using 40% small-sized CFs, a maximum thermal conductivity of 2.89 W/(m·K) was achieved [44]. In another study, when using 15% BN and 15% CF, a thermal conductivity of 1.84 W/(m·K) was obtained [43]. Compared with other similar works (Figure 3c), this study achieved a thermal conductivity of 0.41 W/(m·K) using only 1% CF. In practical applications, we should balance the thermal conductivity enhancement and mechanical strength decline. Increasing the CF content in the PVDF composite tends to sacrifice the mechanical properties of the composite [44].

The selection of the 1%CF@PVDF/LM-5 composite as the target is justified by both thermal performance and material cost considerations. Although the 2%CF@PVDF/LM-5 composite exhibited the highest thermal conductivity, the increase from 1 to 2 wt% of the CFs is associated with declining returns in conductivity enhancement relative to the addition of CFs. Given that CFs are relatively expensive, the cost-effectiveness of using 1 wt% of the CFs is more favorable. Furthermore, at 1 wt% CF loading, the improvement in thermal conductivity is substantial compared to the PVDF/LM-5 composite (Figure 3b), confirming the role of the CFs in reinforcing thermal conduction networks without excessive material usage. This optimized composite balances performance improvement and economic feasibility, making it a potential option for thermally conductive PVDF composites.

### 3.3. Crystallinity and Crystal Structures of Composites

**Figure 4:**
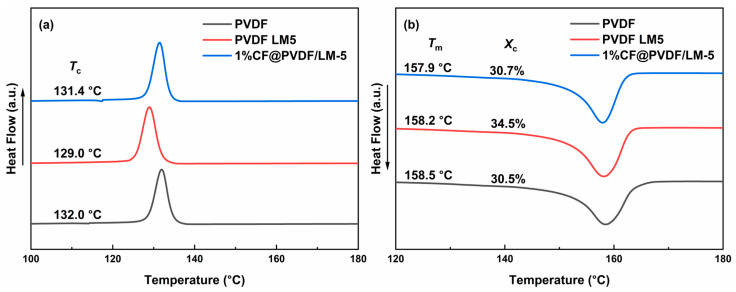
DSC thermal analysis of the PVDF-based composites: (**a**) cooling and (**b**) heating curves.

**Figure 5:**
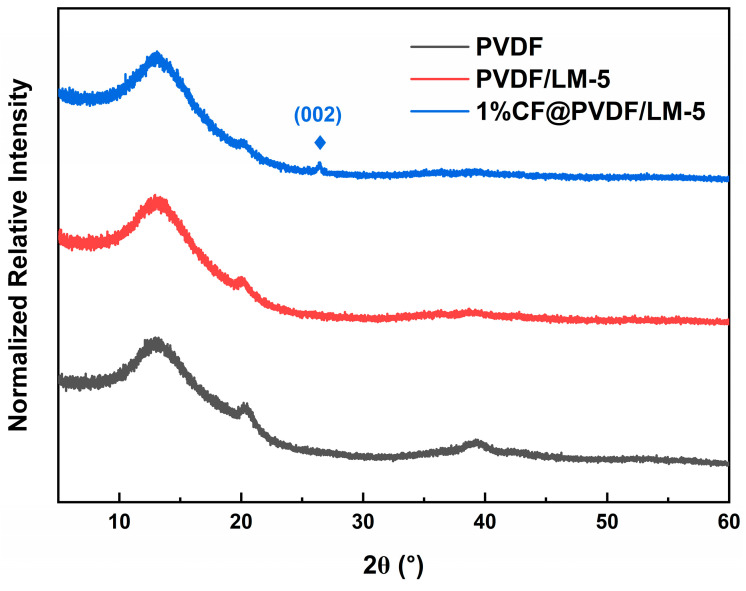
The XRD patterns of the PVDF-based composites.

The crystallinity and crystal structures of the PVDF-based composites were investigated using DSC (Figure 4) and XRD (Figure 5). Figure 4 presents the DSC cooling and heating curves of the pure PVDF, PVDF/LM-5, and 1%CF@PVDF/LM-5 composites. The corresponding melting points (*T*_m_), crystallization temperatures (*T*_c_), and crystallinity (*X*_c_) are listed in Figure 4. The *T*_c_ of the pure PVDF was 132.0 °C, while adding the LMs and the CFs slightly decreased the *T*_c_ of the PVDF-based composites. The PVDF/LM-5 composite showed a lower *T*_c_ of 129.0 °C, caused by the influence of the LMs on the crystallization kinetics. In contrast, the 1%CF@PVDF/LM-5 composite displayed a *T*_c_ of 131.4 °C, which is close to that of the pure PVDF. The improved *T*_c_ suggests that the CF served as an additional nucleating agent, counteracting the impact of the LMs and stabilizing the crystallization behaviors. Both the LMs and the CFs acted as the nucleation sites, but the fiber shape of the CFs enhanced the PVDF crystallization by providing a higher density of nucleation sites.

The heating curves in Figure 4b show that the *T_m_* of the pure PVDF was 158.5 °C. The inclusion of the LMs in the PVDF/LM-5 composite slightly lowered the *T_m_* to 158.2 °C, while the 1%CF@PVDF/LM-5 composite showed a further decrease in *T_m_* to 157.9 °C. These minor differences in the *T*_m_ values suggest no substantial changes in the crystalline structure of the PVDF after filler addition. The nearly identical *T_m_* values indicate that the incorporation of the LMs and the CFs did not significantly disrupt the chain alignment or thermal stability of the PVDF matrix during the melting process. These results imply that the fillers were primarily dispersed physically rather than altering the crystalline structure of the PVDF matrix, assisting in preserving its intrinsic thermal properties. In addition, the *X*_c_ of the pure PVDF was 30.5%, whereas the PVDF/LM-5 composite showed a notable increase to 34.5%. This enhancement in *X*_c_ likely resulted from the LM particles acting as nucleation sites that promoted a higher degree of crystalline order. The *X*_c_ of the 1%CF@PVDF/LM-5 was 30.7%, which was more closely aligned with that of the pure PVDF. This suggests that, while the CFs promoted nucleation, the combined effect with the LMs in the PVDF matrix resulted in a moderate overall crystallinity similar to that of the pure PVDF.

In summary, adding the LMs and the CFs to the PVDF matrix affects the crystallization behaviors and crystallinity of the composites. The LMs lowered *T*_c_ but increased *X*_c_ due to their role as nucleation sites, promoting a higher degree of crystalline order. The incorporation of the CFs partially improved *T*_c_ by acting as additional nucleation agents, stabilizing the crystallization behavior, and maintaining a moderate crystallinity level similar to that of the pure PVDF. The minimal changes in *T_m_* across all composites indicate that the fillers primarily dispersed physically without significantly altering the crystalline structure or thermal stability of the PVDF matrix. Additionally, despite these moderate changes in crystallinity, the significant impact on thermal conductivity (Figure 3) suggests that other factors, such as enhanced filler connectivity, play a crucial role in improving the thermal performance of the composites.

The XRD patterns of the PVDF-based composites (Figure 5) indicate that adding the LMs and the CFs to the PVDF matrix did not significantly alter its crystal structure. The pure PVDF displayed characteristic peaks corresponding to the α-phase [46], which remained predominant in both the PVDF/LM-5 and 1%CF@PVDF/LM-5 composites. This suggests that the incorporation of the LMs and the CFs did not induce substantial phase transitions within the PVDF matrix, maintaining its original crystal structure.

In the 1%CF@PVDF/LM-5 composite, a new peak appeared at approximately 26.4° (002). This peak corresponds to the crystalline structure of the CFs [47], indicating the successful incorporation of the CFs into the composite. Minor peak shifts and broadening were observed at approximately 20.5° and 39.5° for the 1%CF@PVDF/LM-5 composite, which indicate slight interactions between the fillers and the PVDF matrix. However, these effects do not imply a change in the primary crystalline phase of the PVDF matrix. These results suggest that the fillers can enhance properties, such as thermal conductivity, without disrupting the intrinsic crystalline structure of the PVDF.

### 3.4. FTIR Analysis

**Figure 6:**
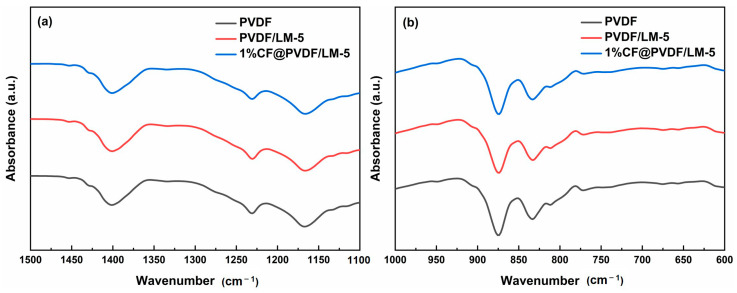
The ATR-FTIR spectra of the pure PVDF and the PVDF composites in different wavenumber ranges: (**a**) 1500–1100 cm^−1^ and (**b**) 1000–600 cm^−1^.

ATR-FTIR spectra of the pure PVDF and the PVDF-based composites in different wavenumber ranges are presented in Figure 6. The characteristic absorption bands of the pure PVDF were observed in both regions across all the composites, including peaks associated with the α-phase at approximately 840 and 760 cm^−1^. These results confirmed the primary crystalline structure of PVDF.

Comparing the spectra of the pure PVDF with those of the PVDF/LM-5 and the 1%CF@PVDF/LM-5 composites, no significant shifts, new peaks, or changes in intensity were observed. This indicates that the addition of the LMs and the CFs did not introduce new chemical bonds or significantly modify the molecular structure of the PVDF matrix. The retained spectral features suggest that both the LMs and the CFs were physically dispersed within the PVDF matrix without altering the intrinsic bonding structure or phase composition of the PVDF matrix.

The absence of noticeable changes in the FTIR spectra supports that the fillers did not chemically interact with the PVDF matrix but instead interacted physically. This finding aligns with the structural stability observed in other analyses (i.e., DSC and XRD), suggesting that the LMs and the CFs enhanced thermal and mechanical properties by forming a stable composite network within the PVDF matrix, rather than by chemically altering it.

Such physical bonding provides a straightforward and scalable method to enhance properties without compromising the inherent chemical stability of polymer matrix [48]. It can avoid potential issues related to polymer degradation, phase transformation, or unwanted side reactions, making it suitable for applications requiring long-term durability [49]. However, the primary limitation of physical bonding is that the effectiveness of property enhancement relies heavily on filler dispersion, interfacial adhesion, and percolation threshold [50,51,52]. Poor dispersion may lead to agglomeration, which can hinder the formation of efficient thermal conductive pathways [8].

In contrast, chemical bonding, such as surface functionalization or grafting reactions, offers the advantage of tailoring interfacial interactions between the polymer matrix and the fillers, potentially leading to stronger bonding and improved compatibility [53]. It can enhance mechanical performance, interfacial thermal transport, and even phase stability. However, chemical bonding often involves complex synthesis processes, additional reagents, and potential alterations to the intrinsic properties of polymer matrix, such as its crystallinity or electrical properties [16]. Additionally, chemical reactions may introduce defects or by-products that can negatively impact material performance [54].

Given these considerations, the selection between physical and chemical bonding depends on the specific application requirements. In this study, a physical bonding strategy was adopted to maintain the chemical integrity of the PVDF matrix, while improving the thermal conductivity through the synergistic combination of the LMs and the CFs. This approach ensures that the desirable properties of the PVDF matrix are maintained, while enhancing the heat transfer performance of the composites.

### 3.5. Mechanical Properties of PVDF/LM/CF Composites

**Figure 7:**
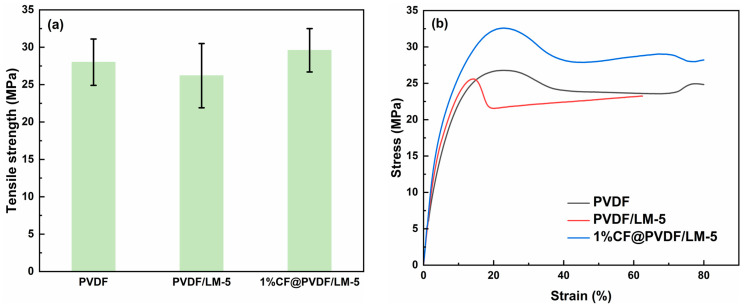
The mechanical properties of the pure PVDF and the PVDF composites: (**a**) tensile strength, (**b**) stress–strain curves.

The mechanical properties of the pure PVDF and the PVDF-based composites are shown in Figure 7. In Figure 7a, the tensile strength of the pure PVDF was 28.0 ± 3.1 MPa. Upon adding 5 wt% of the LMs, the tensile strength of the PVDF/LM-5 composite slightly decreased to 26.2 ± 4.3 MPa. This slight reduction is likely due to the inherently low mechanical strength of the LMs, which acted as a rigid inclusion without reinforcing the PVDF matrix. However, when 1 wt% CF was added into the PVDF/LM-5 matrix (1%CF@PVDF/LM-5), the tensile strength increased to 29.6 ± 2.9 MPa, which was higher than that of the pure PVDF. This increase can be attributed to the high tensile strength and reinforcing ability of the CFs, as the CFs introduced a network structure within the PVDF matrix that effectively distributed applied stress, as observed in the SEM images (Figure 2(c1,c2)).

The SEM images in Figure 2 reveal that the CFs not only provided a reinforcing network but also improved the dispersion of the LM droplets within the PVDF matrix. In Figure 2(c1), the LM droplets were more uniformly distributed in the presence of the CFs, which minimized large voids and stress concentrators that could weaken the PVDF matrix. This uniform distribution and the networked structure of the CFs contributed to an enhanced load-bearing capacity, as the CFs helped bridge stress points within the PVDF matrix, leading to improved tensile strength and the structural cohesion in the PVDF composite.

The stress–strain curves (Figure 7b) further highlight the effect of the LMs and the CFs on the deformation behaviors of the PVDF matrix. The pure PVDF displayed a ductile profile with moderate elongation at break. The PVDF/LM-5 composite showed a reduction in strain at break due to the rigid LM inclusions acting as microcrack initiators under tensile loading. The addition of the CFs in the 1%CF@PVDF/LM-5 composite, however, counteracted this brittleness to some extent. The CFs improved load transfer within the PVDF matrix, mitigating the brittleness introduced by the LMs and supporting a more balanced mechanical response.

Apart from the tensile strength data, the stress–strain behavior (Figure 7b) further highlights the mechanical roles of LM and CF fillers. The pure PVDF exhibited a typical ductile profile with moderate elongation at break, whereas the addition of LMs led to a noticeable decrease in ductility due to the rigidity of LM inclusions and potential microcrack initiation at the filler–matrix interface. In contrast, the 1%CF@PVDF/LM-5 composite showed an improved mechanical response with a more balanced stress–strain curve. The reinforcing effect of CFs helped bridge stress concentration zones and delayed fracture, contributing to improved structural cohesion.

These trends align with SEM fracture surface observations (Figure 2), where the CF-containing composite displayed a more networked and less porous morphology, suggesting improved stress transfer and crack deflection pathways. The combined morphological, structural, and mechanical evidence confirms that incorporating CFs into the LM-PVDF system helps restore or enhance mechanical performance, despite the slight embrittlement introduced by LMs.

Enhancing the thermal conductivity of polymer composites often requires a high content of thermally conductive fillers, and adding fillers may sacrifice the mechanical properties of the polymer. Compared with other related studies with larger amounts of fillers (Table 1), our polymer composite (the 1% CF@PVDF/LM-5 composite) achieved improvements in both thermal conductivity and mechanical property by incorporating much smaller amounts of CF and LM. In addition, the high liquidity issue of the LM as a filler was also overcome by introducing CF as an extra filler.

In summary, the addition of the LMs and the CFs to the PVDF matrix resulted in a composite with enhanced tensile strength, where the CFs served as the primary reinforcing phase. The uniform dispersion of the LM droplets in the presence of the CF and the networked structure of the CFs contributed to a composite with improved load distribution and tensile strength. Although long-term aging behavior was not investigated in this study, the consistent morphology, chemical structure, and mechanical performance indicate that the composite is structurally stable under standard processing and storage conditions.

## 4. Conclusions

This study demonstrated that incorporating LMs and CFs into PVDF significantly enhanced the thermal conductivity and the mechanical strength of the PVDF-based composites. The thermal conductivity of the PVDF/LM-5 composite was 0.35 W/(m·K), representing an improvement of 122%. Adding 1 wt% of the CFs to the PVDF/LM-5 composites (namely, 1%CF@PVDF/LM-5) further raised the thermal conductivity to 0.40 W/(m·K) with an improvement of 154%. This improvement resulted from the formation of continuous conductive pathways enabled by the uniform dispersion of the LMs and the bridging function of the CFs within the PVDF matrix. Mechanical testing showed that the CF addition countered the slight reduction in tensile strength caused by the LMs, achieving a tensile strength of 29.6 ± 2.9 MPa in the 1%CF@PVDF/LM-5 composite, compared to the pure PVDF. Crystal structural analysis confirmed the stability of the crystal structure in the PVDF, with no chemical changes observed between the fillers and the polymer matrix. Practical thermal management applications are currently limited by the lack of long-term thermal cycling and leakage data. In the future, long-term stability, aging, and leakage resistance under elevated temperatures or humidity should be investigated. Furthermore, while mechanical and thermal performance were demonstrated at the lab scale, scaling up the process and ensuring reproducibility remain important challenges. Future work may compare this low-loading strategy with higher loading systems to further understand the trade-offs between thermal performance, mechanical integrity, and processability.

## Figures and Tables

**Table 1:** A comparison of the effects of various fillers on the thermal conductivity and mechanical strength in different polymer composites (the neat polymer matrix is the baseline).

Base Materials	Fillers	Composites	Thermal Conductivity (W/(m∙K))	Thermal Conductivity Improvement	Tensile Strength (MPa)	Tensile Strength Change	Refs
PE	BN	28 wt% BN/PE	0.94	121%	32	−2.7%	[55]
PP/PE	BN	15 wt BN/PP/PE	0.87	264%	28	91.2%	[56]
PP	BN	19.1 vol% BN/PP	4.08	2000%	22.7	8.4%	[57]
EP	Si_3_N_4_/CF/LM	50 wt% Si_3_N_4_/30 wt% CF/55 wt% LM/EP	0.88	389%	4.3	35.1%	[58]
TPU	LM	50 vol% LM/TPU	2.12	909%	1.52	−171%	[59]
PVDF	β-SiCw@SiO_2_	28 wt% β-SiCw@SiO_2_/PVDF	1.78	123%	1.52	22%	[60]
PVDF	CF	40 wt% CF/PVDF	2.89	1700%	34.9	−147%	[44]
PVDF	BN/CF	15 wt% BN/15 wt%CF/PVDF	1.89	1014%	25.4	−26.8%	[43]
PVDF	CF/LM	1 wt% CF@PVDF/LM-5	0.40	154%	29.6	5.7%	This work

PE: polyethylene; BN: boron nitride; PP: polypropylene; EP: Epoxy resin; CF: carbon fiber; LM: liquid metal; TPU: thermoplastic polyurethane; PVDF: poly(vinylidene fluoride).

## Data Availability

The original contributions presented in this study are included in the article. Further inquiries can be directed to the corresponding authors.
